# Simply Applicable Method for Microplastics Determination in Environmental Samples

**DOI:** 10.3390/molecules26071840

**Published:** 2021-03-25

**Authors:** Urška Šunta, Polonca Trebše, Mojca Bavcon Kralj

**Affiliations:** Faculty of Health Sciences, University of Ljubljana, Zdravstvena pot 5, 1000 Ljubljana, Slovenia; urska.sunta@zf.uni-lj.si (U.Š.); polonca.trebse@zf.uni-lj.si (P.T.)

**Keywords:** polyethylene terephthalate, polystyrene, polyvinyl chloride, polyethylene, polypropylene, gas chromatography–mass spectrometry

## Abstract

Microplastics (MPs) have gained significant attention in the last two decades and have been widely researched in the marine environment. There are, however, less studies on their presence, routes of entry, and impacts on the biota in the soil environment. One of the main issues in the study of MPs is a lack of standardized methods for their identification in environmental samples. Currently the most commonly used techniques are thermal desorption gas chromatography–mass spectrometry (GC–MS) methods and pyrolysis followed by GC–MS. In this study, headspace-solid phase microextraction followed by GC–MS is proposed as a simple and widely applicable method for the determination of commonly present polymer MPs (polyethylene terephthalate, polystyrene, polyvinyl chloride, polyethylene, and polypropylene) in environmental samples, for analytical laboratories with basic equipment worldwide. The proposed method is based on the identification of compounds, which are formed during the well-controlled melting process of specific coarse (1–5 mm) and fine fraction (1 mm–100 μm) MPs. The method was upgraded for the identification of individual polymer type in blends and in complex environmental matrices (soil and algae biomass). The successful application of the method in complex matrices makes it especially suitable for widescale use.

## 1. Introduction

Industrial development has accelerated the manufacture and the disposal of plastic materials. Currently, the increased use of single use plastics, for convenience or hygiene (e.g., pandemic), is only a stone in the mosaic of plastic pollution, still it represents a significant contributor to microplastic (MP) pollution. Since the turn of the century there has been a steady rise in scientific interest in MPs in environmental research, though most of it focuses on the MPs in the marine environment. While the impacts of marine MPs have gained scientific and significant media and public attention, there is a paucity of research conducted on the terrestrial environment. This is despite the claim of researchers that MPs are also accumulating on land, especially in agricultural areas. The number of publications concerning MP pollution in soils has risen, however, as the topic has gained scientific attention over the last five years. During this time, it has been found that the mass of MPs in soils may be as much as four times greater when compared to the marine environment [1,2,3]. There is currently a gap in the knowledge of MP pollution in terrestrial systems because of a lack of standardized methods, which are available for the quantification of MPs in soils. Therefore, an exact and effective analysis method for assaying MPs in soil samples is needed [3] and steps toward a simple, widely available, and reliable method for the most abundant polymer types [4]:, polyethylene terephthalate—PET, polystyrene—PS, polyvinyl chloride—PVC, polypropylene—PP, and polyethylene—PE (high density) would be of great interest to the scientific community.

There are numerous routes of entry for MP pollution into the soil environment. Landfills from urban and industrial centers, irrigation with wastewater, lake water flooding, littering roads and illegal waste dumping, and vinyl mulch used in agricultural activities, for example, can introduce MPs, which settle on the surface and penetrate subsoils [5]. The collection of soil samples to test for the representative MPs concentration in the final product is the most important step and can be easily influenced by intrinsic factors (due to heterogeneity of MPs and soil types, depth, and soil-layer and accurate representation of soil status: overall, average, etc.). The soil samples should be chemically and physically pretreated (dried, sieved, floated, filtered, extracted, and digested) [3,6]. Finally, MPs can be identified using non-destructive and destructive methods. Visual methods, such as scanning electron microscope (SEM) are usually combined with spectroscopic techniques to confirm the presence of MPs. Micro-Fourier transformed infrared (m-FT-IR) and Raman spectroscopy are commonly used methods. Destructive methods rely on thermal analytical methods, such as thermogravimetry (TGA), which measure weight loss. Gaseous products may then be analyzed with thermal desorption gas chromatography mass spectrometry (TED-GC–MS) [7,8]; or pyrolysis followed by identification of MPs’ degradation products employing gas chromatography–mass spectrometry (Py-GC–MS) [9,10,11,12,13]. Identification of MPs using destructive methods has been widely exploited for the identification of PET, PS, PVC, PP, and PE. However, each polymer type has specific thermal degradation pathways and products.

PET is polyester with ethylene glycol and dimethyl terephthalate or terephthalic acid as monomer units, which is mainly used in the production of food packaging and beverage bottles. The mechanisms of PET thermal decomposition have been reported to be intramolecular backbiting and chain scission leading to cyclic oligomers, vinyl ester, and acid end-groups. PET thermal decomposition was found to be accelerated in the presence of oxygen and chain scission was found to occur more commonly at 280 °C in the presence of water [14,15]. The main degradation products of PET, found in the thermal degradation studies, were carbon dioxide, acetaldehyde, vinyl benzoate, terephthalic acid, benzaldehyde, divinyl terephthalate, benzoic acid, linear dimers, and cyclic oligomers of up to three monomer units in size [15].

PS is an aromatic polymer, produced by the polymerization of styrene monomer units and is used to produce packaging materials and insulation [16]. Dominated PS thermal decomposition mechanisms are thought to be random and chain-end scissions, followed by depolymerization, intramolecular transfer and bimolecular termination, which lead to the formation of styrene and its larger units (dimers, trimers), aromatics, light hydrocarbons, and some oligomeric fragments [8,14,17,18]. The main degradation products of TED-GC–MS and Py-GC–MS analyses were identified as styrene, 2,4-diphenyl-1-buten (dimer), and 5-hexene-1,3,5-triyltribenzene, 2,4,6-triphenyl-1-hexene (trimers) [7,8,13,18,19,20].

PVC is the most common halogenated polymer, made with the polymerization of vinyl chloride, and currently it is mainly used in the production of construction materials and household accessories [14,16]. Thermal degradation of PVC is reportedly a complex process, generally taking place in two stages [21]. The first stage is the dehydrochlorination process (200–277 °C), where hydrogen chloride (HCl) is eliminated via random scission of secondary chlorine atoms and leaving the unsaturated hydrocarbons behind [14,22]. The second stage (above 300 °C) comprises of the degradation of the double bond hydrocarbon backbone, and following the hydrogen evolvement, further splitting up the unsaturated hydrocarbons into small unsaturated linkages, resulting in the occurrence of volatile saturated and unsaturated, aliphatic and aromatic hydrocarbons, such as benzene and toluene [22,23,24,25]. Py-GC–MS for MPs identification mainly detected aromatic hydrocarbons, such as biphenyl, toluene, naphthalene, fluorene, benzene [10,18,20], while chlorobenzene was only detected with the TED-GC–MS method [19]. The lack of detected chlorinated compounds with these methods could be, according to Krauskopf et al. [10], a consequence of enhanced elimination of the main dehydrochlorination compound, HCl. Chlorinated hydrocarbons, namely 1-chlorododecane and dichlorobenzene, were detected after thermal decomposition of waste plastic pellets at 200 °C in the air and 1-chlorobutane was detected as a VOC from PVC based wrapping films [26,27].

PP and PE are the most commercially produced and used aliphatic polymers. They are obtained using the polymerization of ethylene and propylene, respectively, and their thermal decomposition has been well researched. Hydrocarbons with several structural types and isomers are the main thermal decomposition products of polyolefins, such as PP and PE and this is the main reason why their identification poses a challenge [28]. PP reportedly degrades by random backbone chain scission into a repeating pattern of mainly methylated alkenes and unsaturated hydrocarbons [10,29]. Out of these, the most common and abundant is reported to be a trimer, 2,4-dimethylhept-1-en, as the main product of PP splitting [28,30]. This trimer was also identified in Py-GC–MS and TED-GC–MS analyses of PP, and commonly identified thermal degradation products 2,4,6-trimethyl-1-nonene, 2,4,6,8-tetramethyl-1-undecene, and 2,4,6-trimethyl-1-nonene [7,13,18,19,20].

Despite PE and PP both being polyolefins, the thermal decomposition of PE gives different distribution of components than the decomposition of PP [29,30]. The main mechanism of PE thermal decomposition has been reported to be random chain scission into saturated, monounsaturated, and diunsaturated hydrocarbons [28]. These are, according to Dümichen et al. [31], unique in their formation of distinct patterns of series of triplets of the aliphatic carbons (alka-α,ω-diene, alke-1-en, and n-alkane). Studies using Py-GC–MS for MPs identification and quantification identified characteristic series of n-alkanes, n-alkenes, and n-alkadienes, with the following fragment ions selected for quantification in environmental matrices: *m*/*z* 55 for tridecane homologue [10], *m*/*z* 85 for n-alkanes (eicosane) [19,20], and eicosane with *m*/*z* 83 [20]. For conclusive identification of PE in environmental matrices with TED-GC–MS 1,12-tridecadiene, 1,13-tetradecadiene, 1,14-pentadekadien and 1,15-hexadecadiene, and alkene-alkadiene pairs from C_11_ to C_16_ were chosen [7,31].

The protocols for monitoring MPs are recognized by the ASTM standards, only for the initial step of analytical procedure. In fact, the adopted standards recognize only the sampling and preparation of water samples with a high, medium, and low content of suspended substances [32]. Standards for the identification of MPs with Py-GC–MS [33], infrared and Raman spectroscopy [34], on the other hand, are still under development. Polymers are too diverse (Table 1) to allow their direct identification regardless of the dimension, or type. In addition to their chemical diversity, during the production of plastic products, different additives are added (for example antioxidants, flame retardants, and plasticizers) [35].

The headspace-solid phase microextraction GC–MS (HS-SPME-GC–MS) technique has already been successfully applied to the characterization of polymers and the identification of VOCs from expanded PS [37,38], and on its basis the method for the identification of the most common polymers in MPs was developed.

The aim of our research was to develop a simpler identification method for MPs in complex soil matrices based on conventional sample preparation techniques. To make this method feasible, the SPME technique was coupled to simple instrumentation commonly found worldwide in environmental laboratories (GC–MS, also with lower sensitivity). This was compared to instrumentation not necessarily routinely available to every laboratory, like the Py-GC–MS and the TED-GC–MS [19,39]. This paper proposed the use of HS-SPME-GC–MS, previously employed for monitoring technological processes, to identify the five most common types of MPs (polyethylene (PE), polypropylene (PP), polystyrene (PS), polyvinylchloride (PVC), and polyethylene terephthalate (PET)) found in environmental samples (alluvial soil, organic compost, and algae biomass), specifically soil and water, according to their dimension (1–5 mm and 1 mm–100 μm).

## 2. Results and Discussion

### 2.1. Identification of MPs Coarse Fraction (1–5 mm)

Internal standard, deuterated dimethyl phthalate (DMP-d6; 5 μL) with a fragment ion *m*/*z* 166 and t_R_ = 12.627 min was chosen as an indicator for repeatability of the sample analyses and the performance of the SPME fiber for all polymers as it did not cause interference with other compounds and their proposed fragment ions for polymer identification. The relative peaks’ area ratios of selected characteristic compounds for the identification of PET, PS, PVC, PP, and PE (fraction 1–5 mm), compared to the internal standard ranged from 20% to 366% (Figure S2), making DMP-d6 the appropriate choice for all tested MP types.

#### 2.1.1. Polyethylene Terephthalate

**Figure 1:**
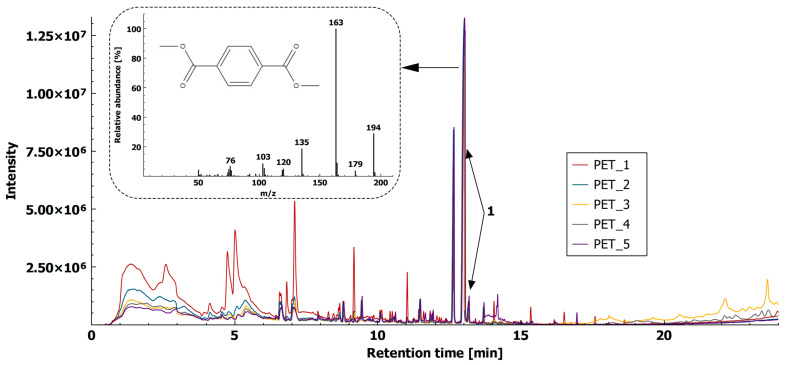
Headspace-solid phase microextraction gas chromatography–mass spectrometry (HS-SPME-GC–MS) extracted ion chromatogram (XIC, *m*/*z* 163) of PET (5 replicates) with mass spectra of dimethyl terephthalate.

In our study, we identified methyl 4-methylbenzoate, methyl 4-ethylbenzoate, and methyl 4-formylbenzoate (t_R_ = 8.775 min, t_R_ = 10.067 min, and t_R_ = 11.461 min, respectively), which were also found using the TED-GC–MS method [8]. PET identification with Py-GC–MS [10,19,20] was based on the presence of dimethyl terephthalate (DMTP), which was also found in this study (fragment ion *m*/*z* 163 at t_R_ = 13.005 min) with HS-SPME-GC–MS. Its presence was additionally confirmed by the synthetized standard DMTP (the synthesis described in Section 3.4.) and matching the two chromatograms, and mass spectra (Figure S3). The reproducibility of the method was expressed as a relative standard deviation (RSD) of five chromatographic peaks for dimethyl terephthalate—DMTP (t_R_ = 13.005 min) and it was found to be below 20% (namely 18.7%) (Figure 1).

#### 2.1.2. Polystyrene

In addition to styrene (t_R_ = 3.214 min) and dimer *trans*(*cis*)-1,2-diphenylcyclobutane (t_R_ = 15.165 min and t_R_ = 15.725 min), reported previously by Dümichen et al. [8] and Krauskopf et al. [10], our method enabled the identification of benzaldehyde (t_R_ = 5.237 min), acetophenone (t_R_ = 6.878 min), 1,3-diphenylpropane (t_R_ = 14.348 min), and benzyl acetophenone (t_R_ = 17.182 min) originating from the thermal decomposition process.

**Figure 2:**
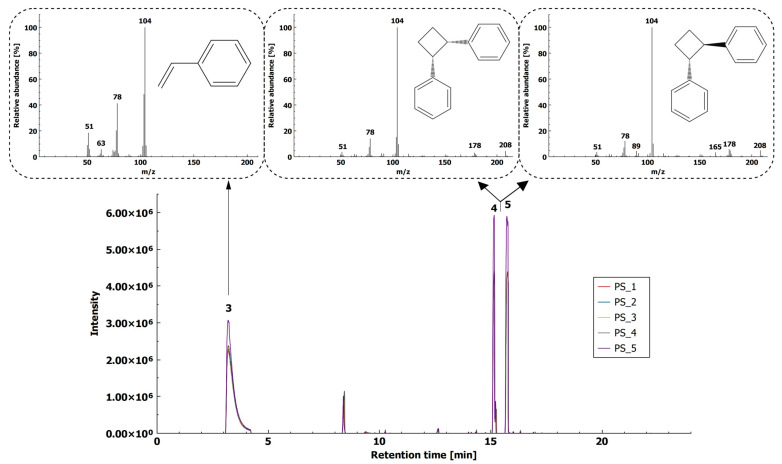
HS-SPME-GC–MS extracted ion chromatogram (XIC, *m*/*z* 104) of PS (5 replicates) with mass spectra of styrene (3) and *trans*(*cis*)-1,2-diphenylcyclobutane (4 and 5).

Styrene was reported to have insufficient specificity as a reliable compound for PS identification in environmental matrices [19], since it can originate from thermal decomposition of chitin and other polymers (e.g., polymethyl methacrylate, PVC), the dimer *trans*(*cis*)-1,2-diphenylcyclobutane (with proposed fragment ion *m*/*z* 104 at t_R_ = 15.165 min and t_R_ = 15.725 min (Figure 2)) was proposed as a more suitable compound for PS identification. The reproducibility of the method, expressed as RSD of five chromatographic peaks for *trans*(*cis*)-1,2-diphenylcyclobutane, was below 15% (12.2% and 13.3% at t_R_ = 15.165 min and t_R_ = 15.725 min, respectively).

Styrene and two dimers were confirmed by matching the chromatograms of MP and the reference particle (Figure S4). The styrene reference compound confirmed the monomer unit in MP chromatograms. Unfortunately, the reference compounds for dimers were not currently commercially available, which was also observed and reported by Krauskopf et al. [10]. Thus, their confirmation was not possible, and the identification of styrene dimers was based on their presence in the reference material and the interpretation of the mass spectra from the NIST library.

#### 2.1.3. Polyvinyl Chloride

**Figure 3:**
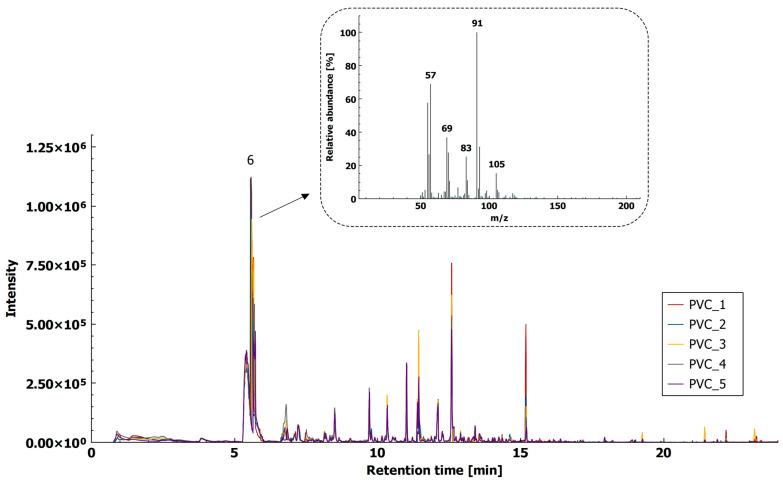
HS-SPME-GC–MS extracted ion chromatogram (XIC, *m*/*z* 91) of PVC (5 replicates) with mass spectra of 1-chlorooctane.

Saido et al. [40] identified 2-chlooctane as a thermal decomposition product of PVC in the presence of dioctyl phthalate, which is a commercial plasticizer used in the production of flexible PVC. Using our HS-SPME-GC–MS method, we were able to detect a number of aromatics, and chlorinated compounds, namely chlorinated aliphatic hydrocarbons. During thermal decomposition of the polymer, the color visually changed from transparent to yellow, indicating the second stage of PVC thermal decomposition was reached [41]. Due to its specificity, a chlorinated hydrocarbon, 1-chlorooctane (t_R_ = 5.58 min) with fragment ion *m*/*z* 91 was proposed for PVC identification with HS-SPME-GC–MS (Figure 3). Beside 1-chlorooctane, other tentatively identified compounds were 2-ethylhexyl acetate (t_R_ = 6.906 min), naphthalene (t_R_ = 8.427 min), dimethyl adipate (t_R_ = 9.290 min), and 2-ethylhexyl chloroacetate (t_R_ = 10.187 min). The reproducibility of the method, expressed as an RSD of five chromatographic peaks for 1-chlorooctane (t_R_ = 5.580 min), was below 32% (namely 31.4%).

Analysis of the reference material, in powder form, using the protocol for coarse fraction identification, also indicated the presence of chlorinated aromatic compounds (benzyl chloride, chloro-methyl styrene, and 1,2,4-trichlorobenzene) and other aromatic compounds (acetophenone, naphthalene, biphenyl, fluorene, and phenanthrene), previously reported as thermal degradation products of Py-GC–MS [10,18,19,20] (Figure S5).

#### 2.1.4. Polypropylene

**Figure 4:**
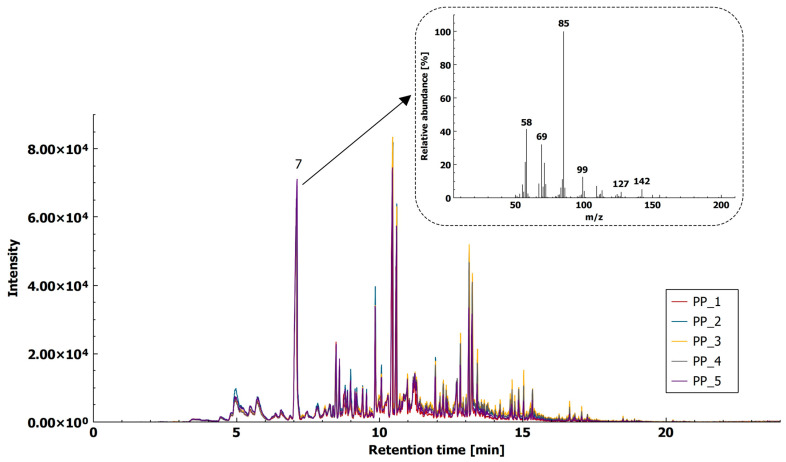
HS-SPME-GC–MS extracted ion chromatogram (XIC, *m*/*z* 142) of PP (5 replicates) with mass spectra for 4,6-dimethyl 2-heptanone (7).

Using the HS-SPME-GC–MS method, we were able to tentatively identify methylated alkanes and methylated ketones (Figure 4). Identification of ketones as decomposition products is attributed to the presence of oxygen during thermal decomposition in air. Beyler and Hirschler [14] state that oxygen affects the mechanism and rate of decomposition, so that the decomposition temperature is reduced by about 73 °C, making it possible for volatilization of PP to take place at 102 °C. The mechanism of oxidative composition is suggested to firstly involve the decomposition of peracids and the subsequent oxidation of primary decomposition products [42]. The proposed fragment ion for PP identification is *m*/*z* 142, with a characteristic compound tentatively identified as 4,6-dimethyl 2-heptanone at t_R_ = 7.108 min (Figure 4). The reproducibility of the method, expressed as an RSD of five chromatographic peaks for 4,6-dimethyl 2-heptanone, was below 15% (namely 12.4%).

In case of PP, method verification was carried out by matching the chromatograms of MP PP and PP reference material, as the reference compound for identification (4,6-dimethyl-2-heptanone) was not commercially available. The overlay of the chromatograms showed a good fit between MP PP and PP reference material in total ion chromatogram (TIC) (Figure S6a) and in extracted ion chromatogram (XIC) at *m*/*z* 142 (Figure S6b).

#### 2.1.5. Polyethylene

**Figure 5:**
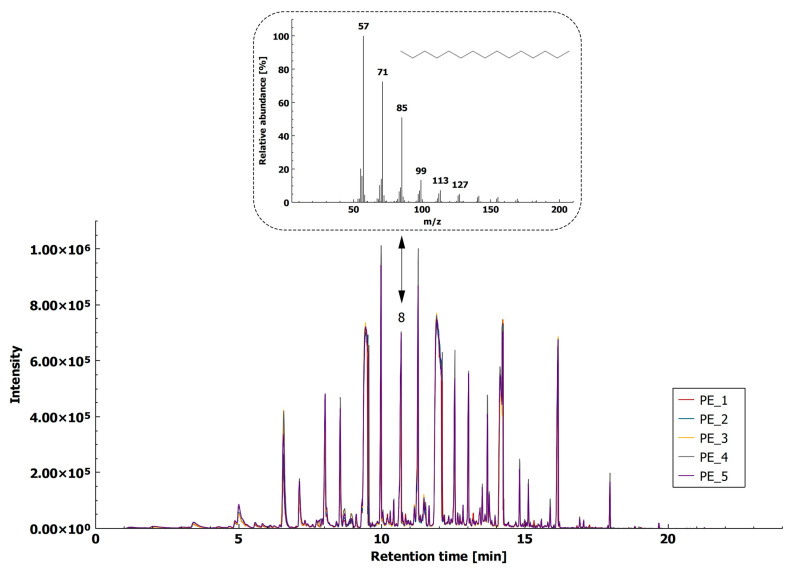
HS-SPME/GC–MS extracted ion chromatogram (XIC, *m*/*z* 85) of PE (5 replicates). Arrow indicates pentadecane(8).

PE, used to develop the method, presented in this study, was high density PE (HDPE), which has a higher melting point as compared to low density PE [14]. In an inert atmosphere, PE crosslinking started at 202 °C, while decomposition started at 292 °C. However, the presence of oxygen was reported to strongly enhance decomposition. Therefore, the decomposition of PE in the presence of air is possible at lower temperatures [14]. For identification of PE using HS-SPME-GC–MS, a fragment ion of *m*/*z* 85 was proposed. XIC of *m*/*z* 85 consists of a series of higher alkanes with a carbon number from C_12_ (t_R_ = 6.546 min) to C_21_ (t_R_ = 17.960 min) (Figure 5, Table S7), and pentadecane (t_R_ = 10.664 min) was chosen for its specificity in the polymer blend containing PE. The reproducibility of the method was expressed as an RSD of five chromatographic peaks for pentadecane, which was 20%.

The presence of pentadecane in PE samples was confirmed by the reference compound and a tight overlay of MPs PE and PE reference material (Figure S8). The selected fragment ion was very common and abundant among hydrocarbons, especially in the environmental samples. Therefore, the suitability of selected characteristic compounds for PE and PP identification was tested in realistic samples containing high hydrocarbon load (soil matrix with added mineral oil and PE and PP MPs, see Section 2.2.2.).

### 2.2. Method Applicability in Environmental Samples

#### 2.2.1. Identification of MPs in the Polymer Blend and Fine Fraction (1 mm–100 μm)

**Figure 6:**
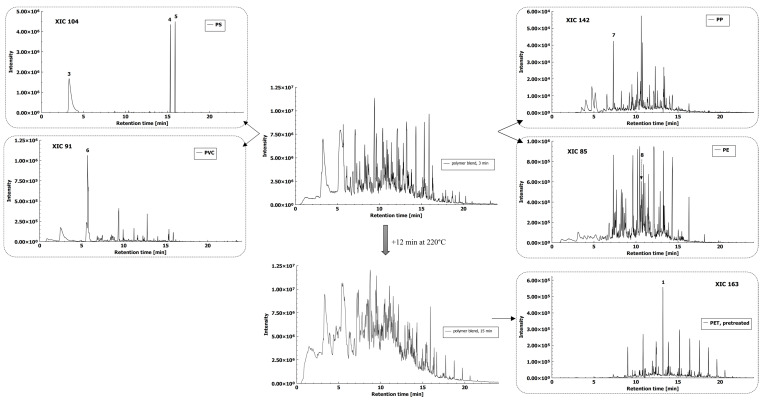
Total ion chromatograms of polymer blend after thermal decomposition at 220 °C for 3 min and 15 min with extracted ion chromatograms of PS (3-styrene, 4 and 5-trans (cis)-1,2-diphenylcyclobutane, PVC (6-1-chlorooctane), PP (7-4,6-dimethyl 2-heptanone), PE (8-pentadecane), and PET (1-dimethyl terephthalate)).

With the use of the described identification method and procedure (see Section 3.2, Section 3.3 and Section 3.4), we were able to identify individual polymers from the polymer blend of coarse fraction (PET, PS, PVC, PP, and PE). PS, PVC, PP, and PE were identified through analysis after thermal decomposition at 220 °C for 3 min. PET was identified through analysis after an additional thermal decomposition at 220 °C for 12 min, 15 min altogether (Figure 6).

MPs with a size range between 1 mm and 100 μm were found to melt faster, therefore thermal decomposition times were adjusted to 1 min for PS, PVC, PP, and PE, and to 3 min for PET. Identification of all MP polymer types was possible with the developed HS-SPME-GC–MS method and XIC comparison with MPs of coarse fraction that were analyzed beforehand (Figure S9). Peak intensities for PS, PE, and PP were in the same range as were particles ranging between 1 and 5 mm, while PVC and PET produced lower intensities of 1-chlorooctane and DMTP, respectively.

#### 2.2.2. Spiked Environmental Matrices

The identification of MPs in complex environmental matrices can be complicated due to the content of various natural organic materials in the samples of water, wastewater, soil, and air [43]. From a qualitative analysis point of view, samples must be pretreated to eliminate the natural organic material and thus avoid interferences of the matrix during the analytical process, and to preconcentrate the MPs content [43,44]. For matrices with low suspended matter content, such as water and air samples, filtration is a suitable pretreatment, while matrices with higher content of natural organic material, such as wastewater and soil samples, must be subjected to more complex pretreatment techniques, like digestion or density separation [45]. Method applicability of HS-SPME-GC–MS was tested in a polymer blend and a complex organic mixture containing algae biomass and organic compost.

A mixture of microalgae, organic compost, and polymer blend to simulate those found in the environment were treated with hydrogen peroxide (H_2_O_2_) to eliminate the organic material before analysis (see Section 3.5). Sample digestion with H_2_O_2_ is a technique, suitable for the digestion of organic material within the matrix, that has a minimum effect on MP damage or further decomposition [46]. Sample pretreatment with digestion, sieving, and/or density separation of MPs is also needed for reliable identification with thermo analytical techniques such as Py-GC–MS and TED-GC–MS, and with spectroscopy techniques such as FTIR and Raman spectroscopy [39,44].

**Figure 7:**
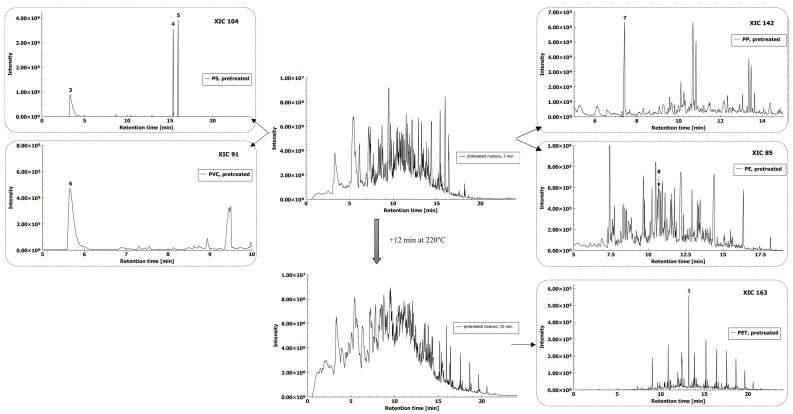
HS-SPME-GC–MS total ion chromatograms of MPs spiked microalgae and organic compost matrix after its pre-treatment and thermal decomposition at 220 °C for 3 min and 15 min with extracted ion chromatograms of identified polymers (polymer-specific compounds: 3–styrene, 4 and 5–trans (cis)-1,2-diphenylcyclobutane, 6–1-chlorooctane, 7–4,6-dimethyl 2-heptanone, 8–pentadecane, and 1–dimethyl terephthalate).

As matrix interferences were minimized by pretreatment of the sample, ions derived from the matrix were not problematic for the GC emergence of selected fragment ions for polymer identification (Figure 7). Each XIC from the mixture was compared to the XIC of individually analyzed MPs (see Section 2.1) and mass spectra in the determined retention times for selected polymer-specific compounds were compared to confirm the MP polymer type.

Synthetic polymers, such as PE, PP, and others analyzed in this study, are derived from petroleum [47]. Therefore, hydrocarbons in the form of alkanes, alkynes, and/or alkenes can be expected thermal decomposition products. The suitability of selected characteristic compounds for PE and PP identification was tested in soil and mineral oil matrix only, and in soil and mineral oil matrix spiked with PP and PE.

After the extraction of PP, identification fragment ion *m*/*z* 142, with the peak at t_R_ = 7.108 min, was not obtained in the soil and mineral oil mixture while there was an evident peak in the spiked mixture. Based on this finding, the suitability of a selected fragment ion for PP identification in environmental samples also containing petroleum-derived compounds was confirmed (Figure S10).

Some problems occurred after the extraction of PE identification fragment ion *m*/*z* 85, as pentadecane (t_R_ = 10.664 min) was also determined in the soil and mineral oil matrix. Overlay of both chromatograms revealed that pentadecane was the last detected higher alkane obtained after analysis of the soil and mineral oil matrix according to the proposed method for MPs identification. However, on the XIC of soil and mineral oil matrix spiked with PP and PE, pentadecane was obtained as previously mentioned, and for PE specific series of higher alkanes, ranging from C_12_ (t_R_ = 6.883 min) to C_20_ (t_R_ = 16.342 min). From these findings, it can be concluded that PE identification in environmental samples with higher load of hydrocarbons, using the HS-SPME-GC–MS method was difficult and less reliable, although it can be done using both identification attributes—pentadecane and the presence of series of higher alkanes.

## 3. Materials and Methods

### 3.1. Microplastic Samples: Type, Shape, and Dimension

Simulated MP samples were prepared from commercially available plastic products of the most abundant polymer types, as suggested by Geyer et al. [4]: PET (mineral water bottle), PS (granulate for laboratory supplies production), PVC (tablet pharmaceutical packaging), PP (granulate for laboratory supplies production), and PE (high density; mineral water bottle caps). PET, PVC, and PE (sizes of 1.9 ± 0.6 mm, 3.7 ± 0.9 mm, and 2.4 ± 0.6 mm, respectively) were manually cut into smaller pieces, whereas PS and PP (sizes of 3.5 ± 0.2 mm and 4.7 ± 0.1 mm, respectively) were left as granules. These represented the so-called “coarse fractions”.

The fine fractions of individual polymer type were obtained by grounding coarse fractions using liquid nitrogen (LN; Messer, Germany) and a ball mill MillMix 20 (Tehtnica, Slovenia) and the following protocol: 6 min cooling in LN, 4 series of milling for 2 min at 35 Hz for PET and at 25 Hz for PS, PVC, PP, and PE with intermediate cooling in LN for 1 min. The obtained particles were sieved through 1 mm and 100 μm stainless steel sieves (Retsch, Germany and Fipis, Slovenia, respectively) to obtain the MP particles ranging between 1 mm and 100 μm, which fit within the general classification terminology of MPs (size range between 1 and <100 µm; [48]).

### 3.2. Experimental Design for Head Space Microextraction of Volatile Organic Compounds Produced from Melted MPs

Sorption of VOCs on the SPME fiber in the headspace (HS) of the sample depends on numerous parameters. The three most important of these are: the type of fiber, the extraction time, and the extraction temperature [49,50]. As the formation of polar VOCs was expected during thermal decomposition of polymers, the mixed phase SPME fiber DVB/CAR/PDMS (50/30 µm) with a length of 20 mm (57299-U, Supelco, Bellefonte, PA, USA) was used. Prior to first use, the fiber was preconditioned at 250 °C for 30 min following the producers’ instructions.

The thermal decomposition process depends on the size, load, and type of the MPs. The amount of MPs sample load was tested in ranges between 10 and 100 mg of MPs, being valid throughout the whole range. Of PET, PS, PVC, PP, or PE 20 ± 5 mg was used in the case of individual polymers and a total of 94 ± 9 mg in the case of the polymer blend was placed into a 20 mL HS vial and sealed with caps with butyl/PTFE septum (Macherey Nagel, Düren, Germany).

The coarse fraction (1–5 mm) samples were placed on the 1st hot plate (IKA, Germany), within the vial, at a temperature of 220 ± 3 °C (testo 926; Testo SE&Co. KGaA, Lenzkirch, Germany) for 3 min (PS, PVC, PP, and PE) and 15 min (PET) to melt. Whereas the fine fraction samples (1 mm–100 μm) were left to melt on the 1st hot plate for 1 min (PS, PVC, PP, and PE) and 3 min (PET). Analyses of individual MP types were conducted in five replicates.

Immediately after thermal decomposition, the vials were transferred in a sand bath (2nd hotplate) with an extraction temperature of 50 ± 2 °C for 20 min. The temperature profiles observed in the HS of the vial during polymer thermal decomposition and VOC’s adsorption onto SPME fiber could be found in the supplementary information (Figure S1). Afterwards, the fiber was inserted into the inlet of the GC with a temperature of 250 °C for 10 min. To prevent any carry-over between analyzed samples, a cleaning procedure with the desorption of compounds from a blank fiber was conducted after each run.

As an internal standard, a solution of deuterated dimethyl phthalate (DMP-d6; Sigma-Aldrich, St. Louis, MO, USA) was used [51]. A standard solution of 50 mg L^−1^, in methanol (HPLC grade, Sigma-Aldrich, St. Louis, MO, USA), was prepared and applied (5 μL), using an ALS syringe (Agilent Technologies Inc., Santa Clara, CA, USA), on the glass wall of the vials containing the MPs sample, directly before the thermal decomposition of the sample. The relative peak area ratios were determined through the analyses of individual polymers of coarse fraction and were calculated by the division of area of the polymer-specific characteristic peak, with the area of the internal standard [52].

### 3.3. Gas Chromatography and Mass Spectroscopy Analysis

After HS-SPME application, desorbed compounds were identified using gas chromatograph 6890 (GC; Agilent Technologies Inc., Santa Clara, CA, USA) coupled with a series of quadrupole mass spectrometer 5973 Network (MS; Agilent Technologies Inc., Santa Clara, CA, USA) operated in the electron impact ionization (EI) mode. Chromatographic separations were carried out on a fused silica 15 m × 0.25 mm × 0.25 μm Rxi-35 Sil MS capillary column (Restek, Centre County, PA, USA). GC operating conditions were as follows: splitless mode; programmed oven temperature from 50 °C (2 min hold) to 270 °C at 10 °C min^−1^. Helium (Messer, Bad Soden, Germany) was used as a carrier gas at a constant flow of 1.0 mL min^−1^. EI occurred at electron multiplier voltage of 1200 V. MS scanned from *m*/*z* 50 to 550 automatically, with an ion source and quadrupole temperature of 230 °C and 150 °C, respectively.

The identification of MP type was based on the structure specific produced compounds after the thermal decomposition and subsequent characteristic fragment ions from MS. Selected structure specific characteristic compounds, adsorbed on SPME, were first tentatively identified using NIST mass spectral reference library (United States National Institute of Standards and Technology, Gaithersburg, MD, USA) for GC–MS by EI and then confirmed with standard reference polymers and standard reference compounds, where possible (see detailed description in Section 3.4).

### 3.4. Standard Reference Materials

Standard polymer particles of PET (granular, bulk density of 1.68 g mL^−1^), PS (beads and bulk density of 1.05 g mL^−1^), PVC (powder, bulk density of 1.4 g mL^−1^), PP (pellets, bulk density of 0.9 g mL^−1^), and PE (pellets, high density, bulk density of 0.95 g mL^−1^) from Sigma-Aldrich (St. Louis, MO, USA) were used as reference polymers. Selected characteristic compounds for polymer identification were confirmed with GC–MS analyses of standard compounds: pentadecane, styrene, 1-chlorooctane, and synthetized dimethyl terephthalate from terephthalic acid (all from Sigma-Aldrich, St. Louis, MO, USA), after dissolving it in methanol (5 mg 10 mL^−1^, HPLC grade, Sigma-Aldrich, St. Louis, MO, USA).

### 3.5. Applicability of Method to Environmental Samples

The applicability of the method was tested on four different complex mixtures. The first mixture was a clean polymer blend (PET, PS, PVC, PE, and PP), the second a fine fraction of each polymer, the third was a polymer blend in a complex organic matrix made from microalgae and organic compost, and the fourth was a mixture composed solely of PE and PP MPs in alluvial soil with mineral oil addition. The polymer blend was selected to determine the applicability of method for identification of individual polymer type in the case of method application in real environmental samples. Furthermore, to examine the possible matrix interference during MPs identification with this method, complex organic matrix, containing microalgae and organic compost was spiked with polymer blend. Microalgae and organic compost were selected to illustrate samples with high organic load. Prior to the analysis, mixture was digested to eliminate organic matter, processed as described in Section 3.2. and Section 3.3. and MPs were identified. Besides, the identification of PP and PE polymer type could be also subjected to interferences due to the presence of mineral oil in soil samples, due to their common natural hydrocarbon load. In order to confirm the decision of selecting fragment ions (85 and 142) for PP and PE identification mineral oil was added to alluvial soil and spiked with PE and PP particles. The particle sizes’ range chosen to test the method applicability was from 1 to 5 mm. A detailed description of the experiments is in the following two paragraphs.

To simulate the process of MP extraction and their subsequent identification from the realistic environmental matrix, domestic compost (1 g) was added to a microalgae culture of 250 mL of *Chlorella vulgaris*. This mixture was spiked with an MP polymer blend (PS, PET, PVC, PP, and PE) with total mass of 89.1 mg. The culture of green microalgae *C. vulgaris* was cultivated beforehand in BBM medium (Phyto technology Laboratories, Rancho Santa Margarita, CA, USA) (prepared according to the manufacturer’s instructions) for 14 days at a room temperature (26 °C), under tubular fluorescent lamps (FLUORA L36W/77, OSRAM, Munich, Germany) with a photoperiod of 16 h/8 h light/dark and was continually stirred at 150 rpm on an orbital shaker (RS-OS 20, Phoenix Instruments, Garbsen, Germany). Domestic compost was dried at 70 °C for 48 h, before its addition to the microalgae culture and the formation of the complex organic matrix.

To separate MPs from the complex microalgae and organic compost matrix, a hydrogen peroxide (H_2_O_2_) digestion and a sieving procedure were applied. The samples were digested using 30% H_2_O_2_ (1:1, *v*/*v*; Merck, Kenilworth, NJ, USA) [46], covered with aluminum foil, and continuously stirred for three days on magnetic stirrers (IKA -Werke, GmbH, Staufen, Germany) at 200 rpm. Afterwards, the digested mixture was sieved through a 500 mm stainless steel sieve (Retsch GmbH, Haan, Germany) and dried in an oven at 65 °C for 24 h. Differences in the mass of undried and dried MPs polymer blend at 65 °C for 24 h were found to be negligible (Δ = 1.6 mg). Obtained particles after filtration were analyzed and MPs were identified according to the method procedure (see Section 3.2 and Section 3.3).

The applicability of PE and PP identification, with selected characteristic compounds and fragment ions, was additionally tested in spiked mixtures of alluvial soil, containing 1% of mineral oil, that is on real samples with a high hydrocarbon load. The characteristics of the alluvial soil, which was used have been previously described in Šunta et al. [53]. In the alluvial soil (1 g) and mineral oil (11 mg) mixture, a portion of 28 mg of PP and 7 mg of PE were added and analyzed according to the method procedure (see Section 3.2 and Section 3.3). Mineral oil used in this case was universal oil for two-stroke engines (Petrol, Ljubljana, Slovenia), which meets the quality requirements of ISO L-EGD/EGC.

## 4. Conclusions

HS-SPME-GC–MS identification of five of the most common polymer types of MPs showed potential to become an acceptable method, since it is based on a simple equipment (SPME fiber, two hotplates, and a sand bath) and GC–MS instrumentation that is commonly found in laboratories worldwide. The method was successfully tested with reference compounds and reference polymers, still some uncertainties in the identification of PE MPs in environmental samples with high load of hydrocarbons can occur due to PE’s chemical structure and thermal degradation products. Robustness of the method and used equipment enable broader use, however it is subjected to manual procedure and MPs quantification depends on the skills of analytical personnel. Besides, some polymers (PS, PET, and PVC) have very high melting temperatures (>180 °C) or are of different shapes, thicknesses, and sizes, which could potentially influence the polymers with lower melting temperatures or cause their incineration and dehydration. If this is the case, a multistep melting procedure with several melting events should be developed. In any case, the applicability of HS-SPME-GC–MS method showed promising results on complex environmental matrices containing MPs.

## Figures and Tables

**Table 1:** Characteristics of the most common polymer types of microplastics (MPs) found in the environmental samples.

Polymer Type	Abbreviation	Formula	Density ^1^ [g/cm^3^]	Melting Point ^1^ [°C]	Glass Transition Temperature ^1^ [°C]
Polyethylene (low and high density)	LDPE	(C_2_H_4_)_n_	0.90–0.92	141	−123–−20
	HDPE		0.95–0.96		
Polypropylene	PP	(C_3_H_6_)_n_	0.85–0.88	179	−15–−3
Polyethylene terephthalate	PET	(C_10_H_8_O_4_)_n_	1.33–1.48	264	65–77
Polystyrene	PS	(C_8_H_8_)_n_	1.04–1.10	242–276	91–128
Polyvinyl chloride	PVC	(C_2_H_3_Cl)_n_	1.38–1.40	220–305	71–107

^1^ [36].

## Data Availability

Data presented in this study are available on request from the corresponding author.

## Supplementary Materials

The following are available online, Figure S1: The temperature profile in the headspace of the vial during sample processing; Figure S2: Relative peaks’ areas ratios of selected characteristic compounds; Figure S3: PET extracted chromatogram overlay of analyzed MP particle, reference particle and compounds; Figure S4: PS extracted chromatogram overlay of analyzed MP particle, reference particle and compound; Figure S5: PVC extracted chromatogram overlay of analyzed MP particle, reference particle and compound; Figure S6: PP extracted chromatogram overlay of analyzed MP and reference particle; Table S7: Thermal decomposition products of PE, identified with HS-SPME-GC–MS; Figure S8: PE extracted chromatogram overlay of analyzed MP particle, reference particle and compound; Figure S9: Extracted ion chromatogram of analyzed MPs in the size range of 1 mm–100 μm; Figure S10: Chromatogram of analyzed PP and PE spiked matrix of alluvial soil and oil.

## Sample Availability

Samples of the compounds are available from the authors.
