# Supplementary material for: Simply Applicable Method for Microplastics Determination in Environmental Samples
Source: Molecules. 2021 Mar 25;26(7):1840. doi: 10.3390/molecules26071840 (PMC8036651; doi:10.3390/molecules26071840)
Supplement: Supplementary file 1 [file molecules-26-01840-s001.pdf]

Supplementary information

Simple and widely applicable method for microplastics determination in environmental samples

Urška Šunta[1], Mojca Bavcon Kralj[2*]

[1]University of Ljubljana, Faculty of Health Sciences, Research Institute, Zdravstvena pot 5, 1000 Ljubljana, Slovenia

[2]University of Ljubljana, Faculty of Health Sciences, Department of Sanitary Engineering, Zdravstvena pot 5, 1000 Ljubljana, Slovenia

Corresponding Author:

Mojca Bavcon Kralj, mojca.kralj@zf.uni-lj.si, 00386 1 300 11 621; Zdravstvena pot 5, 1000 Ljubljana

S1 - S10; 8 pages, 1 tables and 9 figures

Supplement 1: The temperature profile in the headspace of the vial during sample processing

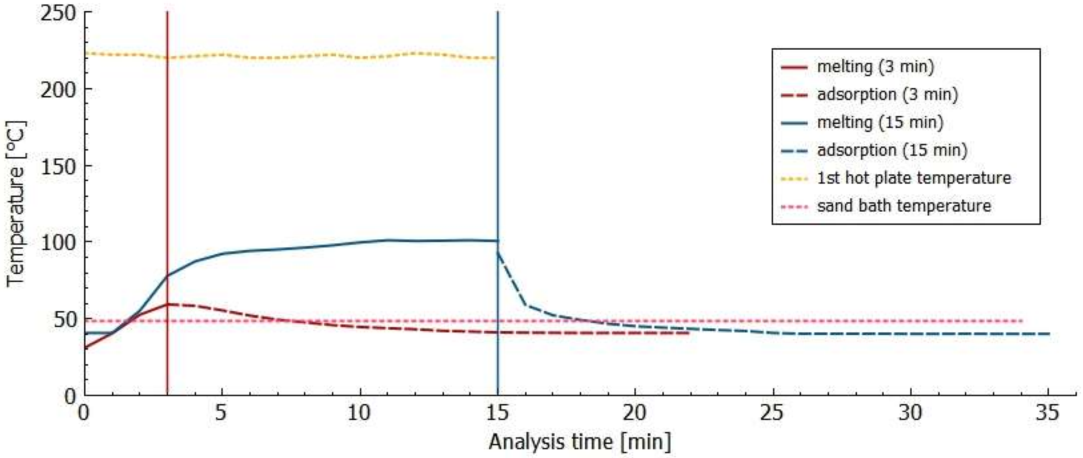

Fig. S1: The temperature profile in the headspace of the vial, during thermal decomposition at 220 °C for 3 min and 15 min, as well as adsorption of VOCs onto SPME fibres in the sand bath.

Supplement 2: Relative peaks' areas ratios of selected characteristic compounds

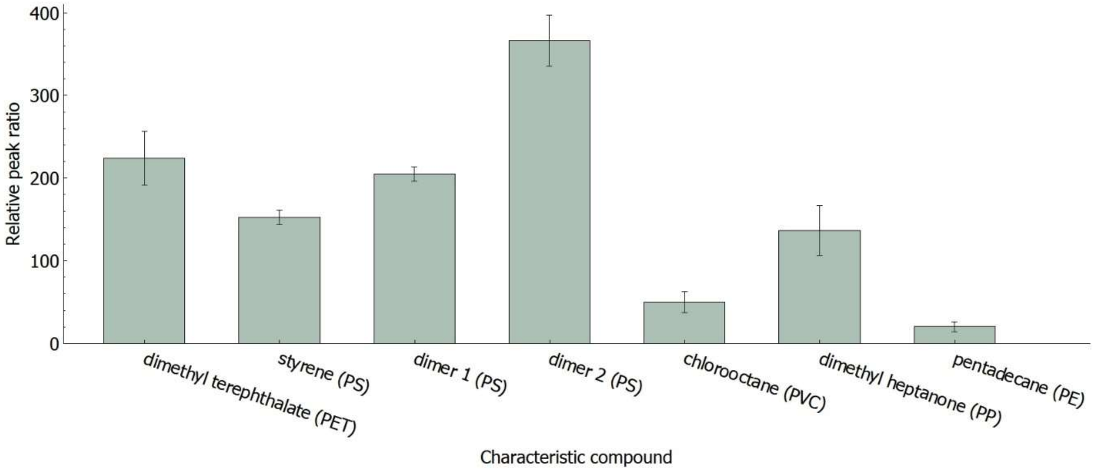

Figure S2: Relative peaks' areas ratios of selected characteristic compounds for identification of PET, PS, PVC, PP and PE (fraction 1-5 mm) compared to internal standard.

Supplement 3: PET extracted chromatogram overlay of analysed MP particle, reference particle and compound

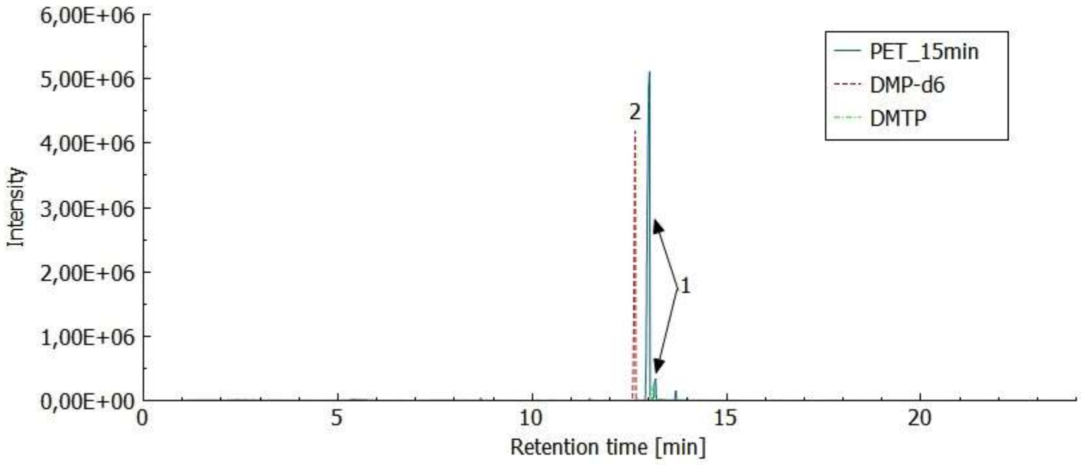

Figure S3: Overlay of extracted ion chromatograms (XIC, *m/z* 163) of analysed MP PET (PET_15min; 1), reference compound (dimethyl terephthalate (DMTP; 1)) and internal standard (DMP-d6; 2)).

Supplement 4: PS extracted chromatogram overlay of analysed MP particle, reference particle and compound

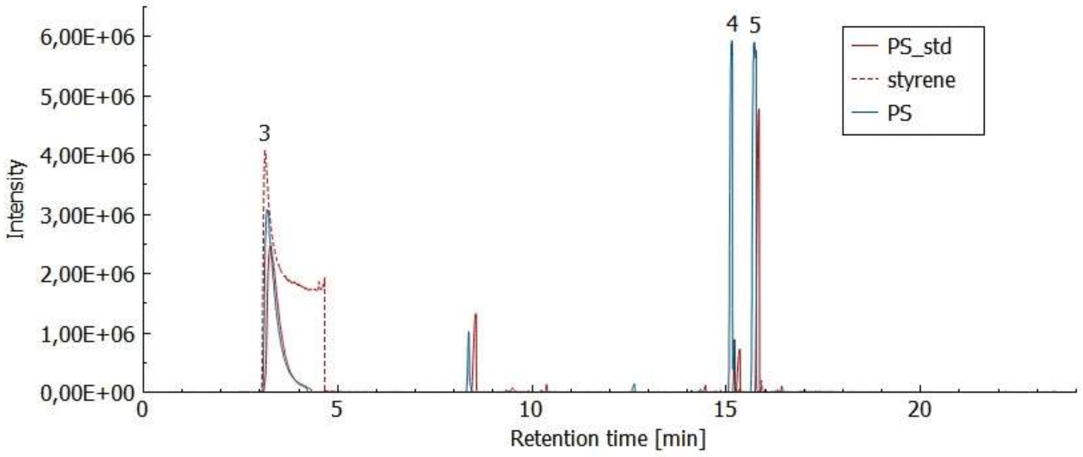

Figure S4: Overlay of extracted chromatograms (XIC, *m/z* 104) of reference material and compound (PS_std and styrene) and analysed MP PS (styrene; 3 and *trans (cis)*-1,2-diphenylcyclobutane; 4,5).

Supplement 5: PVC extracted chromatogram overlay of analysed MP particle, reference particle and compound

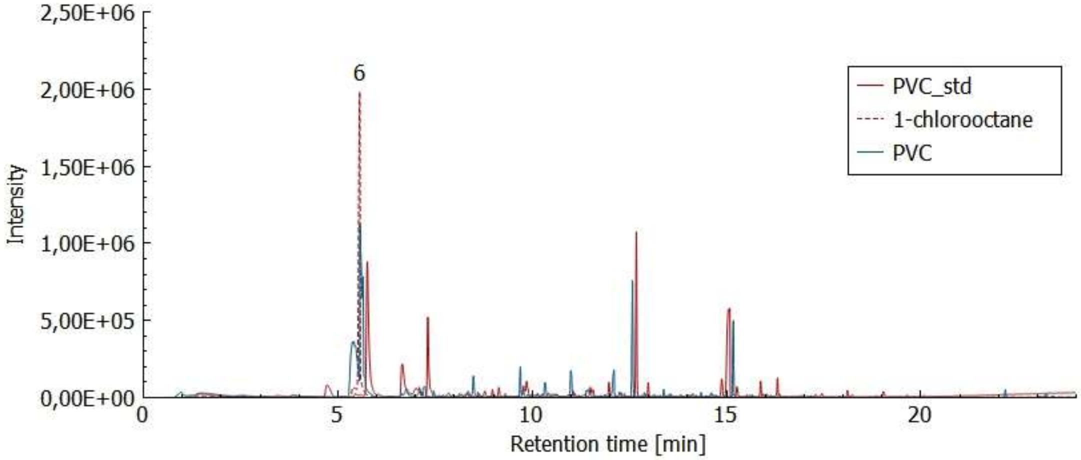

Figure S5: Overlay of extracted ion chromatograms (XIC, *m/z* 91) of reference material and compound (PVC_std and 1-chlorooctane) and analysed MP PVC (1-chloroctane; 6).

Supplement 6: PP extracted chromatogram overlay of analysed MP and reference particle

a)

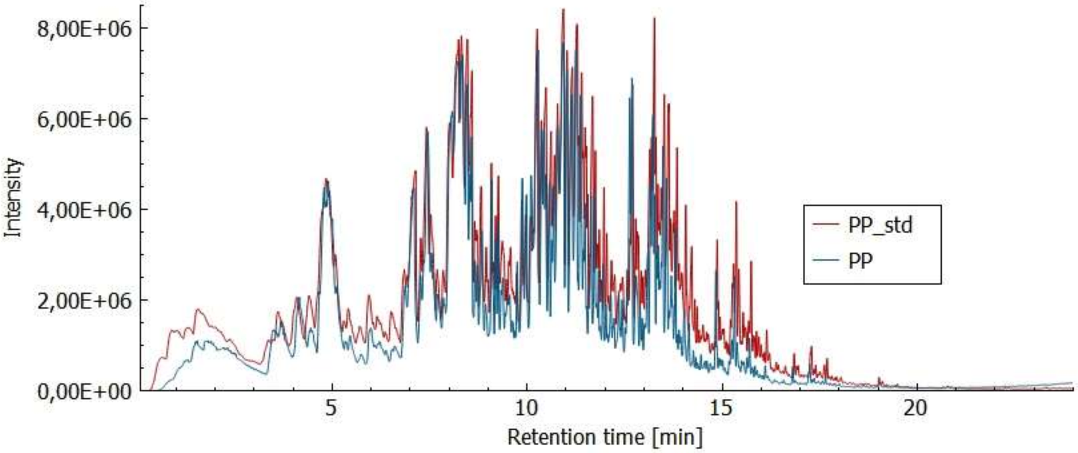

b)

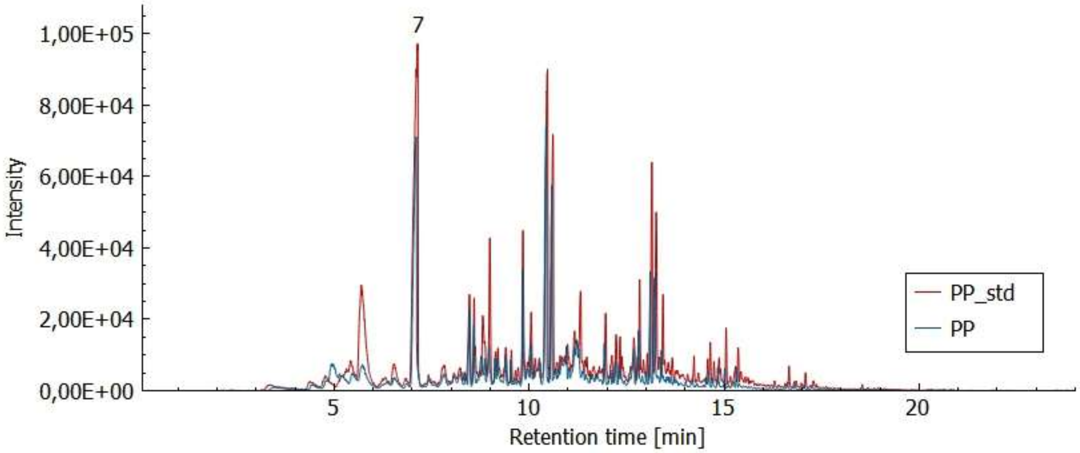

Figure S6: Overlay of total (a) and extracted (b) ion chromatograms (XIC, *m/z* 142) of reference material and analysed MP PP (4,6-dimethyl 2-heptanone; 7).

Supplement 7: Thermal decomposition products of PE, identified with HS-SPME-GC-MS

Retention time	Compound	Molecular formula
6.546 min	Dodecane	$C_{12}H_{26}$
7.998 min	Tridecane	$C_{13}H_{28}$
9.421 min	Tetradecane	$C_{14}H_{30}$
10.664 min	Pentadecane	$C_{15}H_{32}$
11.902 min	Hexadecane	$C_{16}H_{34}$
13.016 min	Heptadecane	$C_{17}H_{36}$
14.222 min	Octadecane	$C_{18}H_{38}$
15.108 min	Nonadecane	$C_{19}H_{40}$
16.131 min	Eicosane	$C_{20}H_{42}$
17.960 min	Heneicosane	$C_{21}H_{44}$

Table S7: Thermal decomposition products of PE, identified with HS-SPME-GC-MS.

Supplement 8: PE extracted chromatogram overlay of analysed MP particle, reference particle and compound

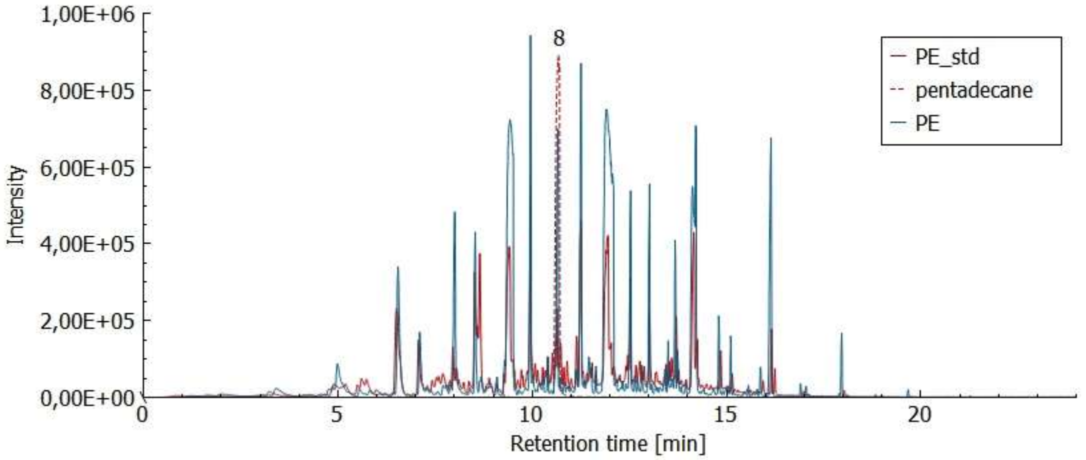

Figure S8: Overlay of extracted ion chromatograms (XIC, *m/z* 85) of reference material and compound (PE_std and pentadecane) and analysed MP PE (pentadecane; 8).

Supplement 9: Extracted ion chromatograms of analysed MPs in the size range of 1 mm-100 µm

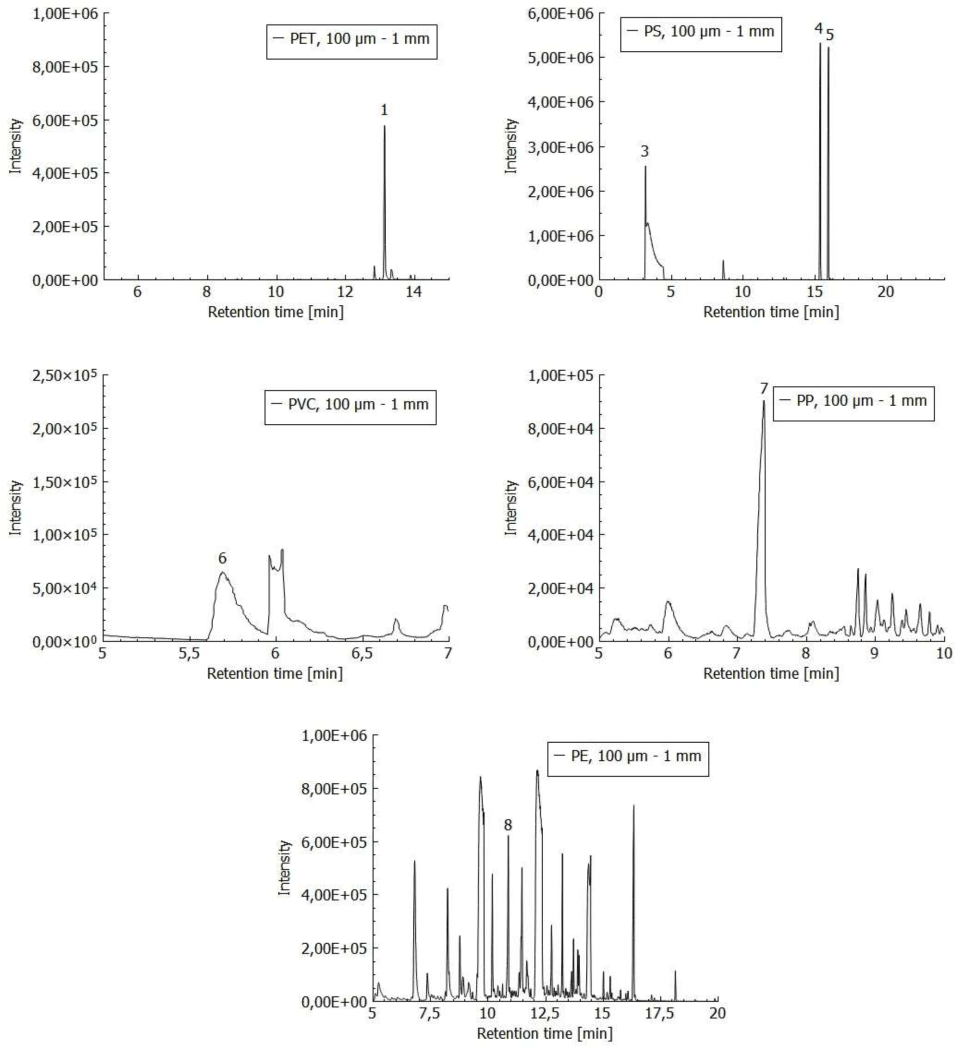

Figure S9: HS-SPME-GC-MS extracted ion chromatograms of MPs in the size range of 1 mm-100 µm for polymer identification with selected polymer-specific fragment ions (polymer-specific compounds: 3 – styrene, 4 and 5 – *trans (cis)*-1,2-diphenylcyclobutane, 6 – 1-chlorooctane, 7 – 4,6-dimethyl 2-heptanone), 8 – pentadecane, and 1 – dimethyl terephthalate).

Supplement 10: Chromatograms of analysed PP and PE spiked matrix of alluvial soil and oil

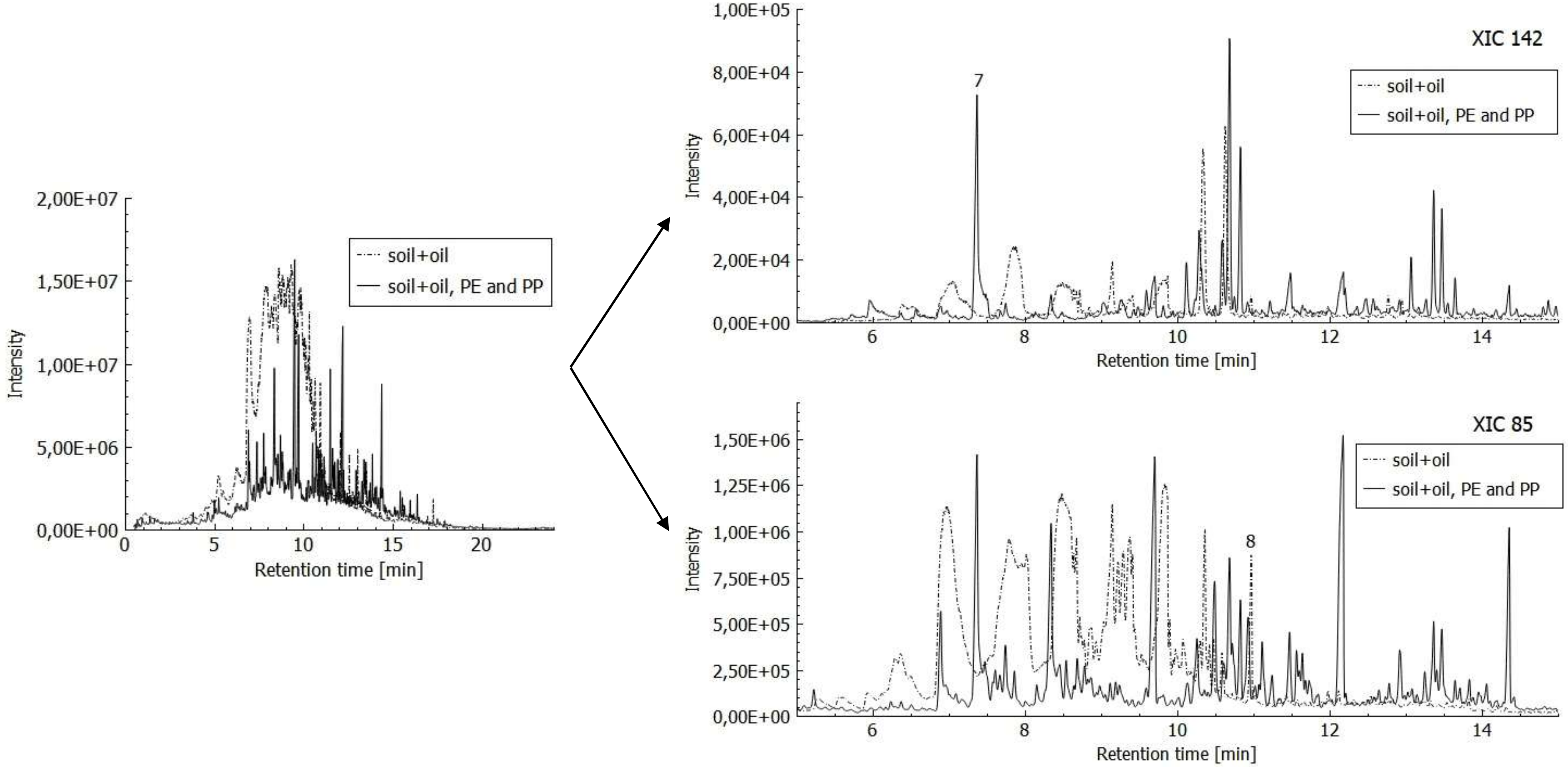

Figure S10: HS SPME GC MS total ion chromatograms of PP and PE spiked matrix of alluvial soil and oil after thermal decomposition at 220 °C for 3 min with extracted ion chromatograms of polymer-specific compounds for identification (7 – 4,6-dimethyl 2-heptanone, 8 – pentadecane).
